# Depressive symptoms are a key determinant of health-related quality of life in ICU survivors with psychological distress

**DOI:** 10.1038/s41598-026-49907-z

**Published:** 2026-05-25

**Authors:** Robert Philipp Kosilek, Nora Schröder, Linda Sanftenberg, Daniela Lindemann, Antina Beutel, Konrad Schmidt, Christian Brettschneider, Hans-Helmut König, Jochen Gensichen

**Affiliations:** 1 Institute of General Practice and Family Medicine, LMU University Hospital, LMU Munich, Nußbaumstr. 5, 80336 Munich, Germany; 2 Institute of General Practice and Family Medicine, Charité University Medicine, Berlin, Germany; 3 Faculty of Health Sciences Brandenburg, Institute of General Practice, Brandenburg Medical School Theodor Fontane, Brandenburg, Germany; 4 Department of Health Economics and Health Services Research, University Medical Center Hamburg-Eppendorf, Hamburg, Germany; 5 German Center for Mental Health (DZPG), Munich/Augsburg, Germany; 6 Institute for Medical Information Processing, Biometry, and Epidemiology (IBE), LMU Munich, Marchioninistraße 15, 81377 Munich, Germany; 7 LMU University Hospital, Medical Clinic IV, Ziemssenstr. 1, 80336 Munich, Germany; 8 Department of General Practice, Clinic of General Medicine, Medical Clinic III, University Hospital Carl Gustav Carus, Technische Universität Dresden, Fetscherstr. 74, 01307 Dresden, Germany; 9 Dritter Orden Clinic, Menzinger Str. 44, 80638 Munich, Germany; 10 School of Medicine, Klinikum Rechts Der Isar, Department of Anesthesiology and Intensive Care, Technical University of Munich, Ismaninger Str. 22, 81675 Munich, Germany; 11 Institute of Psychosocial Medicine, Psychotherapy and Psychoonocology, Jena University Hospital, Stoystr. 3, 07743 Jena, Germany; 12 Clinic of Anesthesiology and Intensive Care Medicine, Brothers of Mercy Hospital Munich, Romanstr. 93, 80639 Munich, Germany; 13 Department of Anaesthesiology, University Hospital, LMU Munich, Marchioninistr. 15, 81377 Munich, Germany; 14 Isarklinikum Anästhesie, Sonnenstr. 24-26, 80331 Munich, Germany; 15 Department of Psychology, University of Konstanz, Universitätsstr. 10, 78464 Konstanz, Germany; 16 Department of Psychiatry and Psychotherapy, LMU University Hospital, LMU Munich, Nußbaumstr. 7, 80336 Munich, Germany; 17 Department of Anesthesiology, Critical Care and Pain Medicine, Harlaching Medical Center, The Munich Municipal Hospitals Ltd, Sanatoriumsplatz 2, 81545 Munich, Germany; 18 Department of Anesthesiology, Critical Care and Pain Medicine, Bogenhausen Medical Center, The Munich Municipal Hospitals Ltd, Englschalkinger Str. 77, 81925 Munich, Germany; 19 Department of Psychiatry and Psychotherapy, Technical University of Munich, Ismaninger Str. 22, 81675 Munich, Germany; 20 Department of Anaesthesiology and Operative Intensive Care, Klinikum Stuttgart, Kriegsbergstr. 60, 70174 Stuttgart, Germany; 21 Clinic for Anesthesiology, Operative Intensive Care and Pain Management, Vivantes Klinikum Neukölln, Rudower Str. 49, 12351 Berlin, Germany; 22 Department of Psychiatry and Psychotherapy of the University Medical Center Hamburg-Eppendorf, Martinistr. 52, 20246 Hamburg, Germany; 23 Clinic of Anesthesiology and Intensive Care Medicine, University Hospital Carl Gustav Carus, Technische Universität Dresden, Fetscherstr. 74, 01307 Dresden, Germany; 24 Department of General Practice/Primary Care, University Medical Center Hamburg-Eppendorf, Haus West 37, Martinistr. 52, 20246 Hamburg, Germany; 25 Department of Psychiatry and Psychotherapy Campus Charité Mitte, Bonhoefferweg 3, 10117 Berlin, Germany; 26 Clinic of Anesthesiology and Intensive Care Medicine, Augsburg University, Stenglinstr. 2, 86156 Augsburg, Germany; 27 Department of Internal Medicine, Klinikum Rechts Der Isar, Technical University of Munich, Ismaninger Str.22, 81675 Munich, Germany; 28 Intensive Care Medicine, Schoen Clinic Bad Aibling Harthausen, Schoen Clinic Group, Balanstr. 71a, 81541 Munich, Germany; 29 Center for Anesthesiology and Intensive Care Medicine, University Medical Center Hamburg-Eppendorf, Martinistr. 52, 20246 Hamburg, Germany; 30 Institute for General Practice and Interprofessional Health Care, University Clinic Tübingen, Osianderstr. 5, 72076 Tübingen, Germany; 31 Intensive Care Unit, Paulinenkrankenhaus gGmbH, Dickensweg 25-39, 14055 Berlin, Germany; 32 Institute of General Practice, Klinikum Rechts Der Isar, Technical University of Munich, Orleansstr. 47, 81667 Munich, Germany; 33 Institute of Psychiatric Phenomics and Genomics, University Hospital, LMU Munich, Marchioninistr. 15, 81377 Munich, Germany; 34 Department of Cardiology, Pneumology and Internal Intensive Care Medicine, Schwabing Medical Center, The Munich Municipal Hospitals Ltd, Kölner Platz 1, 80804 Munich, Germany; 35 Department of Internal Intensive Care, University Clinic Tübingen, Osianderstr. 5, 72076 Tübingen, Germany; 36 Institute of Medical Statistics, Computer and Data Sciences, Jena University Hospital, Bachstr. 18, 07743 Jena, Germany; 37 Fresenius University of Applied Sciences, Limburger Str. 2, 65510 Idstein, Germany; 38 Institute of Diagnostic and Interventional Radiology, University Hospital Frankfurt, Theodor-Stern-Kai 7, 60596 Frankfurt Am Main, Germany; 39 MSH Medical School Hamburg University of Applied Sciences and Medical University, Am Kaiserkai 1, 20457 Hamburg, Germany; 40 Department of Anesthesiology and Intensive Care Medicine, Charitè University Medicine, Charitéplatz 1, 10117 Berlin, Germany

**Keywords:** Critical illness, Intensive care units, Post-traumatic stress disorders, Depression, Health-related quality of life, Diseases, Health care, Medical research, Psychology, Psychology, Signs and symptoms

## Abstract

Survivors of critical illness frequently experience persistent impairments in health-related quality of life (HRQoL), with psychological symptoms contributing substantially to this burden. The relative contribution of co-occurring depression, anxiety, and post traumatic stress symptoms remains insufficiently understood. To address this gap, we conducted a cross-sectional analysis of pre-randomization data from the PICTURE randomized controlled trial, a multicenter study of a brief primary care–based psychological intervention for post-traumatic stress disorder symptoms following critical illness, including 319 intensive care unit survivors. Clinical, demographic, and mental health assessments were obtained after ICU discharge. Latent profile analysis, random forest modeling, and quantile regression were applied to identify determinants of HRQoL measured by the EuroQol Five-Dimension Five-Level (EQ-5D-5L) index and visual analog scale (VAS). The mean EQ-5D-5L index was 0.71 (SD 0.27; median 0.81) and the mean EQ VAS score was 60.7 (SD 19.4; median 60.0), indicating considerable overall impairment. Depression, anxiety, and post-traumatic stress symptoms showed substantial overlap and formed four distinct symptom profiles associated with specific functional impairments. Screening positive for depression on the 2-item Patient Health Questionnaire (PHQ-2) with ≥ 3 points was associated with a median reduction of -0.13 (95% CI -0.19 to -0.07) on the EQ-5D-5L index and -12.45 points (95% CI -17.93 to -6.96) on the EQ VAS, exceeding clinical and demographic predictors. These findings indicate that depressive symptoms are a major determinant of impaired health related quality of life among intensive care survivors with psychological distress and support routine brief depression screening in post-intensive care follow up.

Trial registration: ClinTrials.gov: NCT03315390 (Registration date: 2017-10-20); German Clinical Trials Register: DRKS-ID: DRKS00012589 (Registration date: 2017-10-17).

**Supplementary Information:**

The online version contains supplementary material available at 10.1038/s41598-026-49907-z.

## Introduction

Advances in intensive care have shifted the challenge from survival to survivorship, with many patients living for years with new or worsened problems after critical illness^1,2^. Treatment in an intensive care unit (ICU) saves lives, but also exposes patients to intense physical and psychological stressors^3^. Many survivors face a difficult recovery, marked by intrusive memories, depression, anxiety, cognitive dysfunction and physical impairments^4^, which may be further exacerbated by pre-existing comorbidities^5^.

Post-intensive care syndrome (PICS) encompasses a broad spectrum of cognitive problems, physical deconditioning, and neuropsychiatric sequelae^2^. Systematic reviews consistently demonstrate lasting reductions in health-related quality of life (HRQoL) following critical illness^6–8^. The high prevalence of psychological problems constitutes a substantial disease burden, with meta-analyses estimating that approximately one in five ICU survivors report post-traumatic stress disorder (PTSD) symptoms and roughly one in three report clinically important anxiety or depression^9–11^. These conditions frequently overlap, forming a spectrum of psychological distress that rarely fits into single diagnostic categories^12^.

Despite substantial evidence, critical knowledge gaps remain. First, although HRQoL is markedly reduced in ICU survivors, the specific factors driving this impairment, particularly the relative contribution of co-occurring mental health problems, are insufficiently understood^13^. Second, evidence for post-ICU follow-up programs remains inconclusive, particularly regarding patient-important outcomes such as HRQoL^14–16^. Third, these gaps in knowledge contribute to a broader clinical problem: psychological needs are often insufficiently addressed after ICU discharge, particularly within primary care follow-up, limiting opportunities to generate evidence-based interventions^17^.

Understanding the determinants HRQoL after critical illness is crucial for several reasons^18,19^. Clinically, identifying high-risk patients enables targeted interventions that could prevent or mitigate long-term disability. Economically, addressing modifiable risk factors could reduce the substantial healthcare utilization and societal costs associated with post-intensive care syndrome. Methodologically, establishing key predictors is essential for designing and evaluating interventions aimed at improving patient-important outcomes^20^.

Here, we present a secondary analysis of the PICTURE trial, a randomized study evaluating a brief primary care intervention for patients with PTSD symptoms following critical illness^21^. The rationale for this analysis stems from the need to move toward actionable knowledge on patient-important outcomes with regard to post-ICU mental health. We investigate how mental health, sociodemographic, and clinical factors relate to HRQoL at study entry. Using flexible, data-driven methods, we aim to identify the relative contribution of co-occurring conditions, highlighting which factors are most strongly associated with HRQoL. Additionally, we explore whether the Patient Health Questionnaire—2 (PHQ-2), a brief two-item depression screener, could efficiently identify patients with clinically meaningful HRQoL impairments, providing evidence for a feasible approach that could be incorporated into post-ICU and primary care workflows.

## Results

### Cohort profile

A total of 319 participants were enrolled, with a mean age of 57.7 years (SD 12.7) and 60.8% males. The leading ICU admission diagnosis was cardiovascular disease (40.4%) with a median ICU treatment duration of 8 days (IQR 4–18) and a mean SOFA score of 9.5 (SD 3.9). Overall, the cohort exhibited moderate PTSD symptom severity (mean PDS-5 score 30.6, SD 13.3), alongside significant burden of depressive symtoms (mean PHQ-9 9.6, SD 4.8) and anxiety symptoms (mean OASIS 6.4, SD 4.5). The median EQ-5D-5L index was 0.8 (IQR 0.6–0.9) and the mean EQ-5D-5L VAS was 60.7 (SD 19.4). Meaningful impairments, defined as a score ≥ 2, were common across EQ-5D domains, most notably in pain/discomfort (77.1%, n = 246) and anxiety/depression (70.5%, n = 225). Full baseline characteristics are provided in Table 1.

**Table 1:** Baseline characteristics (N = 319).

Participant characteristics	Summary statistics
Gender(male), % (No.)	60.8% (194)
Age (years), mean (SD)	57.7 (12.7)
Education (CASMIN levels), % (No.)	
Low (1a-1c)	26.3% (84)
Intermediate (2a-2c)	42.3% (135)
High (3a-3b)	26.6% (85)
N/A	4.7% (15)
Main ICU diagnosis (ICD-10), % (No.)	
I (Cardiovascular disease)	40.4% (129)
J (Respiratory disease)	13.8% (44)
U (Other: COVID-19)	7.2% (23)
C (Neoplasms)	6.9% (22)
K (Gastrointestinal disease)	6.0% (19)
Other	25.7% (82)
Emergency admission (vs. elective), % (No.)	30.1% (96)
ICU treatment duration (days) (N = 312), median (IQR)	8.0 (4.0–18.0)
SOFA score (N = 270), mean (SD)	9.5 (3.9)
Polypharmacy (≥ 5 medications), % (No.)	58.9% (188)
PDS-5 score, mean (SD)	30.6 (13.3)
PTSD (PDS-5 score ≥ 36), % (No.)	33.2% (106)
PHQ-9 score, mean (SD)	9.6 (4.8)
Depression (PHQ-9 ≥ 10), % (No.)	49.5% (158)
OASIS score, mean (SD)	6.4 (4.5)
Anxiety (OASIS ≥ 8), % (No.)	40.1% (128)
EQ-5D-5L VAS (N = 318), mean (SD)	60.7 (19.4)
EQ-5D-5L VAS (N = 318), median (IQR)	60.0 (50.0–75.0)
EQ-5D-5L index, mean (SD)	0.71 (0.27)
EQ-5D-5L index, median (IQR)	0.81 (0.61–0.91)
EQ-5D-5L domains: Any problems (≥ 2), % (No.)	
Mobility	50.8% (162)
Self-Care	27.3% (87)
Usual Activities	61.8% (197)
Pain / Discomfort	77.1% (246)
Anxiety / Depression	70.5% (225)

### Mental health comorbidity and symptom profiles

**Fig. 1:**
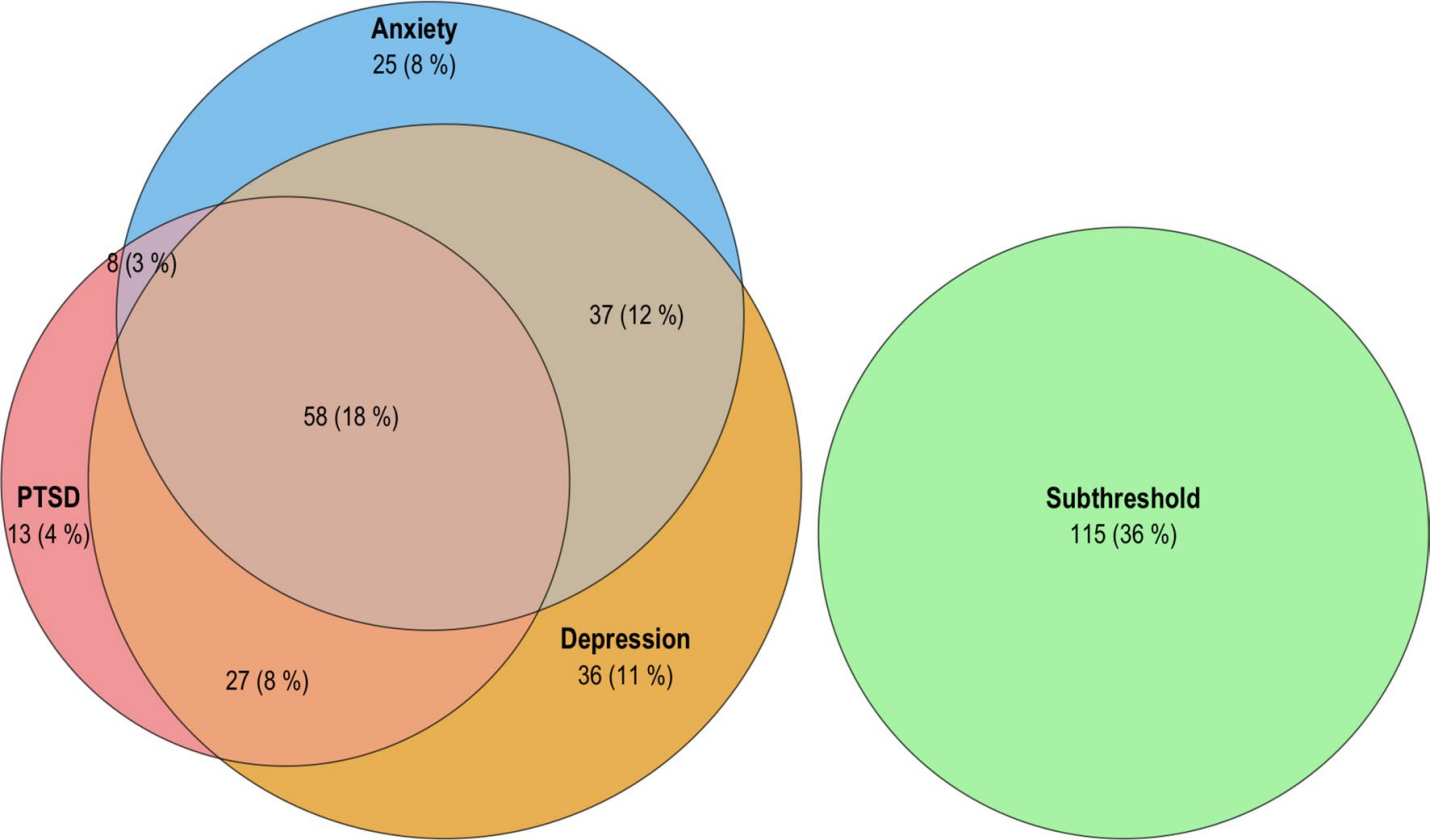
Venn diagram illustrating the overlap of clinically significant PTSD (PDS-5 ≥ 36), depression (PHQ-9 ≥ 10), and anxiety (OASIS ≥ 8) symptoms among ICU survivors at baseline (N = 319). The subthreshold group includes participants below all clinical cutoffs.

**Fig. 2:**
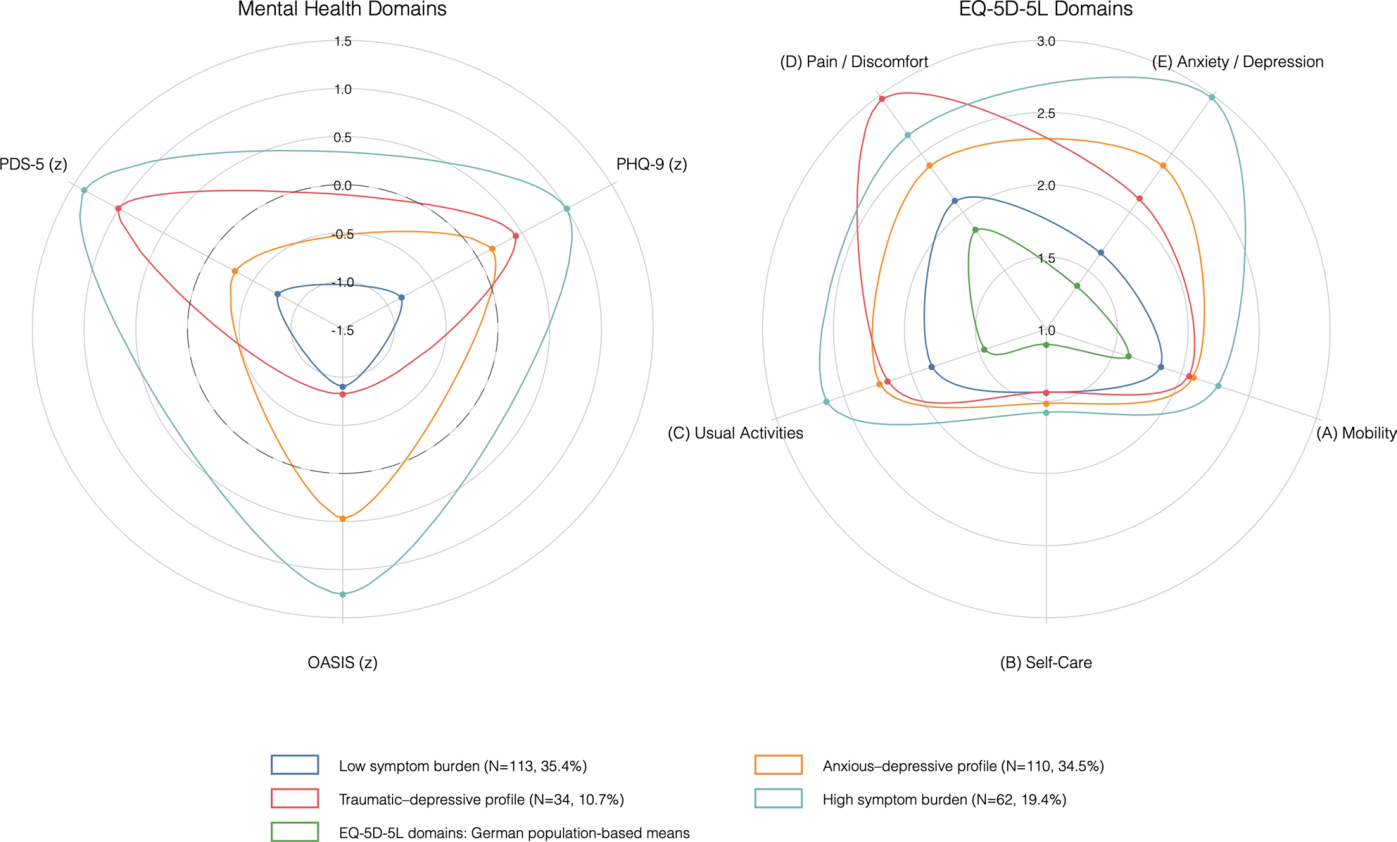
Symptom profiles and corresponding quality of life impairments. Radar plots showing four latent symptom profiles, mapped to standardized mental health scores (left panel: PDS-5, PHQ-9, OASIS z-scores) and EQ-5D-5L domain scores (right panel) . Higher values indicate greater impairment. EQ-5D-5L population means (green) are included for reference, based on published data (Grochtdreis et al. 2019).

Baseline comorbidity of PTSD, depression, and anxiety was high (Table 1). Using established clinical cut-offs (PDS-5 ≥ 36, PHQ-9 ≥ 10, OASIS ≥ 8), 18% met criteria for all three conditions, 23% for two, 23% for one, and 36% were below thresholds (Fig. 1). In the latent profile analysis of PDS-5, PHQ-9, and OASIS scores, model fit indices supported a four-class solution (AIC 6056.48 and BIC 6124.26), which showed improved fit compared with the three-class model (AIC 6105.85; BIC 6158.56) and no meaningful improvement with the five-class model (AIC 6054.01; BIC 6136.84). The Lo–Mendell–Rubin test favored four over three classes (p < 0.001) but not five over four classes (p = 0.326), and the four-class model had the highest entropy (0.78). The resulting mental health profiles were labeled by score distributions: “low symptom burden” (35.4%), “anxious-depressive” (34.5%; elevated PHQ-9 and OASIS, moderate PDS-5), “traumatic-depressive” (10.7%; high PDS-5 and PHQ-9, low OASIS), and “high symptom burden” (19.4%; elevated on all three). Full summary statistics of baseline variables by profile are provided in the Supplement (eTable 1); demographic and clinical variables did not differ meaningfully between profiles. Figure 2 shows mean standardized mental health domain scores (left panel) and mean EQ-5D-5L domain scores (right panel) over the retrieved profiles. The high symptom burden profile showed pronounced impairment across all EQ-5D-5L domains. The anxious-depressive and traumatic-depressive profiles displayed intermediate patterns, with elevations in anxiety/depression or severe pain/discomfort, respectively. Self-care impairment was notably low. All profiles were associated with lower HRQoL than population norms ^22^.

### Determinants of health-related quality of life

**Fig. 3:**
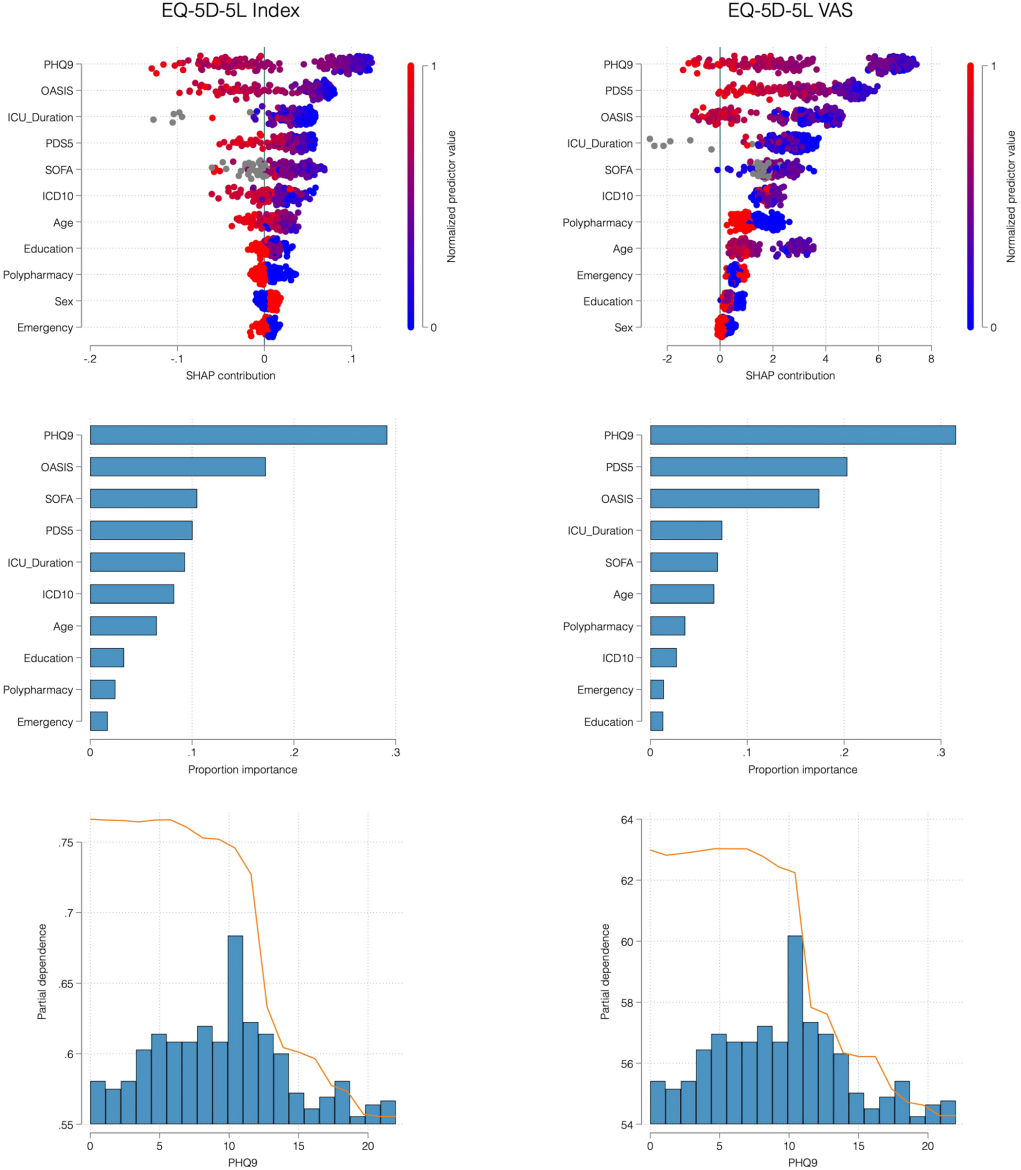
Determinants of health-related quality of life: Random forest results. SHAP (top) and variable importance (middle) plots from random forest models show the relative contribution of baseline variables to EQ-5D-5L index (left) and VAS scores (right). Depressive symptoms (PHQ-9) were the strongest predictor, with partial dependence plots (bottom) illustrating a sharp decline in quality of life above the clinical PHQ-9 threshold of 10 points.

We used random forest regression to identify variables associated with HRQoL. Model performance was assessed using five-fold cross-validation, yielding an RMSE of 0.24 and R^2^ of 0.24 for the EQ index, and an RMSE of 18.4 and R^2^ of 0.09 for the EQ VAS. Across both outcomes, depressive symptoms (PHQ-9) consistently ranked as the most important variable, while the remaining mental health or clinical variables contributed comparatively little to the models. Figure 3 summarizes the random forest results for both EQ-5D-5L outcomes. The PHQ-9 emerged as the leading predictor in variable importance rankings. The partial dependence plots in the bottom panel show the relationship between PHQ-9 scores and predicted HRQoL outcomes, adjusting for all other variables. There is a marked drop at the clinical threshold of ≥ 10, indicating a non-linear relationship, with HRQoL remaining relatively stable at lower PHQ-9 scores but declining steeply once moderate depressive symptoms are present. We verified these findings with quantile regression (τ = 0.5) using the same covariates (Supplement, eTable 2). PHQ-9 z-scores showed the strongest independent associations with both the EQ index (β = –0.06, 95% CI –0.09 to –0.03, p < 0.01) and VAS (β = –5.73, 95% CI –8.62 to –2.84, p < 0.01), while clinical measures again showed only modest associations. Together, these analyses indicate that depressive symptom burden is more strongly associated with quality-of-life impairment than other mental health, clinical or demographic variables.

### Joint Impact of mental health symptom profiles on health-related quality of life

To examine the combined impact of PTSD, depression, and anxiety on HRQoL, we used latent symptom profiles as predictors in quantile regression models (Table 2). Compared to the low symptom burden reference category, participants with high symptom burden had substantially lower EQ index scores (adjusted median difference –0.17, 95% CI –0.23 to –0.10) and VAS scores (–14.18, 95% CI –21.37 to –6.99). The traumatic-depressive profile also showed notable reductions (index: –0.15, 95% CI –0.24 to –0.05; VAS: –12.64, 95% CI –21.01 to –4.28), while the anxious-depressive profile showed moderate associations (index: –0.08, 95% CI –0.13 to –0.03; VAS: –2.96, 95% CI –9.02 to 3.11). Adjusting for age, sex, and education led to only modest attenuations.

**Table 2:** Mental health impact on health-related quality of life.

	EQ-5D-5L Index		EQ-5D-5L VAS	
	Univariate	Adjusted†	Univariate	Adjusted†
Model 1: Symptom Profiles				
Low symptom burden	Ref	Ref	Ref	Ref
	-	-	-	-
Anxious-depressive profile	-0.08***	-0.08***	-5.00*	-2.96
	[-0.14—-0.03]	[-0.13—-0.03]	[-10.89—0.89]	[-9.02—3.11]
Traumatic-depressive profile	-0.14***	-0.15***	-20.00***	-12.64***
	[-0.23—-0.05]	[-0.24—-0.05]	[-27.68—-12.32]	[-21.01—-4.28]
High symptom burden	-0.18***	-0.17***	-20.00***	-14.18***
	[-0.25—-0.10]	[-0.23—-0.10]	[-26.57—-13.43]	[-21.37—-6.99]
Model 2: PHQ-2 Screen (≥ 3 P.)				
PHQ-2 ≥ 3	-0.14***	-0.13***	-20.00***	-12.45***
	[-0.21—-0.07]	[-0.19—-0.07]	[-24.85—-15.15]	[-17.93—-6.96]

Quantile regression models (τ = 0.5) with multiple imputation (m = 25) and robust standard errors (N = 319). Coefficients represent differences in the conditional median with 95% confidence intervals in brackets.

*p < 0.10, **p < 0.05, ***p < 0.01. † Adjusted for sex, age and education.

### Association of core depressive symptoms with health-related quality of life

Finally, we assessed whether the PHQ-2, a brief screener for core depressive symptoms, could capture a similar magnitude of HRQoL differences as those observed for the more complex symptom profiles (Table 2). Participants screening positive on the PHQ-2 (≥ 3 points) had markedly lower EQ-5D-5L index scores (adjusted median difference –0.13, 95% CI –0.19 to –0.07) and VAS scores (–12.45, 95% CI –17.93 to –6.96) compared to those screening negative. Notably, these effect sizes are comparable to those seen in the high symptom burden and traumatic–depressive latent profiles, indicating that the PHQ-2 efficiently captures much of the overall impact of mental health burden on HRQoL.

### Sensitivity analysis

There were only a few observations at the bounds (4.7% for the EQ index and 1.25% for the VAS), supporting standard OLS regression as a comparator frequently used in HRQoL research (Supplement, eTable3). On the EQ index, OLS produced slightly larger deficits for a positive PHQ-2 screen (adjusted mean difference –0.17, 95% CI –0.23 to –0.11), consistent with lower-tail skew. For the EQ VAS, estimates were similar (− 10.82, 95% CI –15.03 to –6.60).

## Discussion

In this study, we analyzed clinical, demographic, and mental health variables to identify factors associated with HRQoL in ICU survivors with PTSD symptoms. We found a high prevalence and substantial overlap of PTSD, depression, and anxiety. Across analyses, depressive symptom burden was most strongly associated with reduced HRQoL, exceeding effects of clinical or sociodemographic variables. We identified four latent mental health profiles – low symptom burden, anxious-depressive, traumatic-depressive, and high symptom burden – with a clear gradient of associated HRQoL impairments. The PHQ-2, a brief two-item screener of core depressive symptoms, captured nearly the same magnitude of impairments as those observed through more sophisticated modeling in the latent profiles with higher symptom burden. These findings suggest that depressive symptoms are a key mental health determinant of impaired HRQoL after critical illness and support routine use of brief depression screening instruments such as the PHQ-2 in post-ICU care.

Our findings align with evidence that survivors of critical illness frequently experience persistent and co-occuring symptoms of PTSD, depression, and anxiety, which collectively diminish HRQoL^23–30^. EQ-5D-5L values from an independent German ICU-survivor cohort closely mirror ours, providing external validation^31^. The median utility decrement of approximately –0.13 on the EQ index we observed for depressive symptoms aligns with or exceeds the impact reported in population-based studies of major chronic conditions such as cancer, cardiovascular disease, and diabetes, which typically produce utility reductions in the range of 0.05–0.15^32–35^. This places depression among ICU survivors on par with other serious chronic illnesses in its impact on quality of life. Domain-level analysis showed high rates of impairment in pain/discomfort and anxiety/depression, while self-care was less frequently affected, consistent with previous research^22,23^. Notably, our cohort’s mean EQ index (0.71) and VAS (60.7) were well below German population norms (index 0.88, VAS 71.6)^22^. Even participants with low symptom burden scored below reference (mean index 0.81, VAS 65.6), while the high symptom burden group, which comprised about 20% of the cohort, scored lower again (mean index 0.60, VAS 52.7). These findings indicate significant and persistent quality of life deficits among survivors of critical illness with psychological distress, even when mental health symptoms are mild. A multicenter ICU cohort was used to define a minimal clinically important difference (MCID) for the EQ-5D-5L index as ≥ 0.08^36^, while a systematic review reported MCIDs of 0.065 (IQR 0.057) for the index and 9.0 points (IQR 5.0) for the EQ VAS^37^. In our study, a positive PHQ-2 screen was associated with a median EQ-5D-5L index decrement of –0.13 and a median EQ VAS reduction of –12.45, both exceeding these established thresholds. These findings highlight the substantial impact of depressive symptoms on health-related quality of life, affecting both societal preference–based utility captured by the index and patient self-rated health reflected in the VAS, and provide strong support for routine brief depression screening in this setting. Latent profile analyses revealed distinct, graded symptom clusters, with profiles combining depression and post-traumatic stress symptoms yielding the worst outcomes. In reviewing the literature, we did not find prior ICU-survivor studies that jointly modeled post-traumatic stress, depression and anxiety symptoms and linked these profiles to quality-of-life outcomes; most prior work focuses on individual conditions or clinical features. Outside ICU populations, however, the comorbidity of PTSD and depression is consistently associated with worse functioning across physical, mental, and social domains of health-related quality of life, which aligns with our findings and supports the plausibility of this profile^38–41^.

Post-ICU research has taken a strong interest in PTSD as a consequence of critical illness^9^, alongside other important sequelae that occur within the umbrella term of post-intensive care syndrome. Yet in our cohort, depressive symptoms showed the strongest association with HRQoL, consistent with evidence that depression after ICU is common, persistent, and frequently comorbid with PTSD^9,11,26^. Depressive symptoms may be downstream of post-traumatic stress symptoms or arise from other common post-ICU stressors such as grief over functional loss, chronic pain, role disruption, or interpersonal strain^42^, and their broad impact on mood, energy, and daily functioning may explain why they were associated with reductions in both the EQ-5D index and the EQ VAS. Interestingly, post-traumatic stress symptoms were more strongly associated with the EQ VAS than the EQ-5D-5L index, possibly reflecting that they primarily affect subjective well-being in a way better captured by patient self-rated VAS scores, whereas the EQ index, based on societal preferences, might be less sensitive to these experiences. We build on prior work by demonstrating that depressive symptomatology may be the primary driver of HRQoL when comorbidity is considered, suggesting that depression might serve as an initial transdiagnostic target in stepped, patient-centred care approaches. Our results highlight the need to systematically address mental health alongside physical rehabilitation during post-ICU follow-up^14,29,43^. Relative preservation of self-care in domain-level findings also suggests that assessing only activities of daily living may miss psychological distress. The brief PHQ-2 screener can capture meaningful HRQoL impairments, requires no specialist training, and integrates readily into primary care, ICU follow-up clinics, and digital pathways^44^. Early pilot data suggest that embedding psychiatric assessment into post-ICU clinics is both feasible and well-accepted^45^. In the traumatic–depressive group, pain/discomfort exceeded other profiles despite comparable clinical parameters, suggesting a distinct pain-anchored phenotype. Unmeasured ICU pain exposure and associated memories may have contributed to post-traumatic stress symptoms beyond depressive burden^46–49^. The signal is supported by evidence showing concordance between the EQ-5D-5L pain item and detailed pain measures in ICU survivors^50^. Guideline-based ICU pain management and brief pain screening with stepped post-ICU care are plausible targets with regard to PTSD outcomes and warrant prospective evaluation in future trials. Although our findings support depression screening in post-ICU follow-up, they reflect the association between HRQoL and depressive symptoms at later recovery time points typical of primary care follow-up; ICU or post-acute clinicians assessing HRQoL closer to discharge should consider that other factors, such as acute illness severity or early functional impairments, may have a higher relative importance earlier in recovery. In summary, our findings support the ongoing shift in post-ICU care and research: from a traditional focus on physical recovery to a broader approach that emphasizes mental health support and patient-important outcomes^17,18,43,51,52^.

Strengths of this analysis include comprehensive clinical and mental health assessment in a well-characterized ICU-survivor cohort, methods suited to skewed HRQoL data, and latent profile analysis capturing comorbidity beyond single diagnoses. Limitations include the cross-sectional design, which limits causal interpretation. Although our sample included a diverse multi-center cohort, eligibility required at least moderate baseline PTSD symptoms, limiting generalizability to post-ICU populations without substantial mental health impairment. The variable timing of trial baseline assessments relative to ICU discharge may further reduce generalizability to ICU survivors at different stages of recovery. Both mental health and HRQoL were self-reported, introducing potential reporting bias. Even though regression models capture overall disease burden using several clinical variables, residual confounding from unmeasured comorbidity or premorbid physical and mental health status cannot be excluded. Because the EQ-5D-5L includes an anxiety/depression domain, some conceptual overlap with the PHQ-9 and OASIS cannot be excluded; however, the EQ-5D index reflects impairments across broader domains of functioning, and the detailed symptom scales were used to derive latent mental health profiles, capturing substantially more complex constructs than the single EQ-5D anxiety/depression item. Finally, HRQoL is a complex, multidimensional construct influenced by a broad range of medical, psychological, and social factors, and the modest R^2^ values observed in regression models likely reflect determinants not captured in the present analysis.

In conclusion, we found substantial overlap of depression, anxiety, and post-traumatic stress symptoms in a sample of ICU survivors with psychological distress, with distinct symptom profiles and related impairments across functional domains. Depressive symptoms were the strongest determinant of reduced health-related quality of life, outweighing clinical and demographic factors, with a utility loss comparable to that seen in other major chronic diseases. These findings support routine brief depression screening in post-ICU care.

## Methods

### Study design and population

This analysis uses pre-intervention data from the PICTURE study, a multicenter randomized controlled trial of a brief psychological intervention for PTSD symptoms after critical illness, conducted in German primary care between 2018 and 2022. The study protocol was approved by the ethics committee of the Medical Faculty of LMU Munich (#17–436), and the trial was conducted in accordance with the Declaration of Helsinki and relevant guidelines and regulations. Participants in the intervention arm received three sessions of narrative exposure therapy (NET) delivered by the GP, while the control group received guideline-concordant usual care across three GP consultations; the primary outcome was improvement in PTSD symptoms at six months. Full details on study design, methodology, and interventions are available in the published trial protocol and primary outcome reports^21,53^. Here, we report a baseline cross-sectional analysis of HRQoL as a secondary outcome. Participants were recruited through collaborating hospitals and primary care practices. Eligible patients were adult ICU survivors aged 18–85 years with significant critical illness, defined by ICU admission with respiratory support and a SOFA score ≥ 3, and at least moderate PTSD symptoms, indicated by a PDS-5 score of 15–70^54–56^. Exclusion criteria included severe physical or concurrent mental health conditions that precluded informed consent or regular participation in the trial and follow-up assessments. Of those screened, 319 participants met the inclusion criteria, provided informed consent, and completed the baseline assessment.

### Measures

In the PICTURE trial, patients were screened for PTSD symptoms at a median of 7 (IQR: 3–17.5) days post-ICU discharge for eligibility, with full pre-randomization baseline assessments completed at a median of 225 (IQR: 149–394) days post-ICU. We collected baseline sociodemographic and clinical covariates after ICU discharge via structured interview and chart review. Sociodemographics included age, sex, and education coded by CASMIN levels^57^. The main clinical variables covered the index ICU stay (primary ICD-10 diagnosis, length of stay, treatment modalities, SOFA score) and current medication intake.

Psychological distress was assessed using three validated self-report instruments: the Post-Traumatic Diagnostic Scale for DSM-5 (PDS-5) for PTSD, the Patient Health Questionnaire (PHQ-9) for depression, and the Overall Anxiety Severity and Impairment Scale (OASIS) for anxiety. The PDS-5 is a 20-item measure of PTSD symptoms—in the past month plus four supplementary questions (items score 0–4, total 0–80, ≥ 36 indicates PTSD), based on DSM-V criteria^54,55^. The PHQ-9 assesses depression over the past two weeks across nine symptoms (items score 0–3, total 0–27, ≥ 10 indicates depression) and is commonly used in primary care research^58^. We additionally calculated PHQ-2 scores, a validated brief screener consisting of the first two PHQ-9 items capturing core affective symptoms (anhedonia and depressed mood) with a cut-off at ≥ 3^44^, to examine whether this brief and easily applicable screening measure could identify patients with clinically meaningful impairments in HRQoL. The OASIS comprises five items measuring the severity and functional impact of anxiety over the past week (items score 0–4, total 0–2, ≥ 8 indicates anxiety)^59^.

HRQoL was assessed using the EQ-5D-5L, which measures five dimensions: mobility, self-care, usual activities, pain/discomfort, and anxiety/depression, each rated on a five-level scale^60^. The EQ-5D-5L produces two summary measures: the index score, which reflects health status across five domains weighted according to societal preferences, and the VAS (visual analog scale), which captures the patient’s overall self-rated health on a scale from 0 (worst imaginable health) to 100 (best imaginable health). The index provides a standardized, population-based valuation of health states, while the VAS represents the individual’s direct subjective assessment of their current health. The EQ index score was calculated using the official German value set^61^, with possible values ranging from states rated as “worse than death” (minimum –0.661), to 0 (death), up to 1 (full health), following the EuroQol Group’s manual^62^. To enable meaningful comparison, we additionally drew reference values from German population norms reported by Grochtdreis et al. in 2019^22^.

### Statistical analysis

Analyses were performed using Stata 19.0 (StataCorp, TX, USA). Baseline characteristics were summarized using descriptive statistics appropriate to each variable’s distribution. The overlap of PTSD, depression, and anxiety, defined by clinical cut-offs, was tabulated and visualized as a proportional Venn diagram using the *eulerr* package^63^ for R Statistical Software (v4.4.2; R Core Team 2024). To characterize mental health profiles within the overlap, we fit a latent profile analysis (LPA) using PDS-5, PHQ-9, and OASIS scores as continuous indicators with Gaussian family/identity link^64^. Because these symptom domains are known to be highly correlated in post-ICU populations, LPA was used to characterize their co-occurrence patterns rather than modeling them as independent predictors. Model fit was assessed based on information criteria (AIC, BIC), Entropy, and the Lo-Mendell-Rubin test. Stratified mean scores were calculated for the resulting solution and visualized in a radar plot using the *spiderplot* package for Stata^65^. Because the EQ-5D-5L index (and, to a lesser extent, VAS) is bounded and left-skewed, standard linear regression may produce biased or unrepresentative results. To address this, we employed random forest modeling^66^, which accommodates complex, nonlinear relationships in skewed data, alongside quantile regression at τ = 0.5^67^, which estimates associations at the conditional median. To provide estimates comparable to those commonly reported in HRQoL research, we additionally estimated mean effects using OLS linear regression as a sensitivity analysis. Robust Huber/White standard errors were used throughout^68^. We examined associations between sociodemographic, clinical, and mental health variables and HRQoL (EQ-5D-5L index and VAS) in two steps. First, random forest regression was conducted using the H2O machine learning framework for Stata^69^, with hyperparameters optimized via grid search for root mean squared error (RMSE) with five-fold cross-validation. Model performance was evaluated using cross-validated RMSE, reflecting the average prediction error, and the coefficient of determination (R^2^), indicating the proportion of variance in the outcome explained by the model. The final model produced Shapley additive explanation (SHAP) beeswarm plots, variable importance plots, and partial dependence plots. Second, findings were verified by median quantile regression with the same variable set. Finally, the association of both LPA-derived symptom profiles and core depressive symptoms based on the PHQ-2 screener with HRQoL was estimated in median quantile regression models, both unadjusted and adjusted for age, sex, and education, capturing baseline vulnerability. Missing data in regression models was handled via multiple imputation (*m* = 25) with chained equations, with regression results pooled according to Rubin’s rules^70^.

## Supplementary Information

Supplementary Information.

## Data Availability

The analytical dataset, which includes de-identified patient data, is available in the research data repository of the Ludwig-Maximilians-University of Munich “Open Data LMU” and can be accessed at https://data.ub.uni-muenchen.de/557/. Access to the dataset is subject to our data use agreement, and further details can be found in the repository documentation. For enquiries about data use, potential collaborations or related projects, interested researchers are encouraged to contact the principal investigator of the study.

## Abbreviations

AIC: Akaike information criterion
BIC: Bayesian information criterion
CASMIN: Comparative analysis of social mobility in industrial nations
CI: Confidence interval
EQ-5D-5L: EuroQol five-dimension five-level
EQ VAS: EuroQol visual analog scale
HRQoL: Health-related quality of life
ICD-10: International classification of diseases, 10th revision
ICU: Intensive care unit
IQR: Interquartile range
OLS: Ordinary least squares regression
OASIS: Overall anxiety severity and impairment scale
PHQ-2: Patient health questionnaire-2
PHQ-9: Patient health questionnaire-9
PDS-5: Post-traumatic diagnostic scale for DSM-5
PICS: Post-intensive care syndrome
PTSD: Post-traumatic stress disorder
RMSE: Root mean squared error
SD: Standard deviation
SHAP: Shapley additive explanation plots
SOFA: Sequential organ failure assessment
