# Supplementary material for: Depressive symptoms are a key determinant of health-related quality of life in ICU survivors with psychological distress
Source: Sci Rep. 2026 May 25;16:16148. doi: 10.1038/s41598-026-49907-z (PMC13201743; doi:10.1038/s41598-026-49907-z)
Supplement: Supplementary file 1 — Supplementary Information. [file 41598_2026_49907_MOESM1_ESM.pdf]

eTable 1. Baseline Characteristics by Mental Health Symptom Profiles

Symptom profile	Low symptom burden	Anxious-depressive profile	Traumatic-depressive profile	High symptom burden	*p* †
N (%)	113 (35.4%)	110 (34.5%)	34 (10.7%)	62 (19.4%)	
Gender (male), % (No.)	64.6% (73)	57.3% (63)	70.6% (24)	54.8% (34)	0.31
Age (years), mean (SD)	58.03 (13.30)	57.64 (12.47)	59.97 (10.00)	55.90 (13.21)	0.49
Education (CASMIN levels), % (No.)					0.56
Low (1a-1c)	20.4% (23)	26.4% (29)	29.4% (10)	35.5% (22)	
Intermediate (2a-2c)	46.0% (52)	41.8% (46)	35.3% (12)	40.3% (25)	
High (3a-3b)	30.1% (34)	27.3% (30)	29.4% (10)	17.7% (11)	
N/A	3.5% (4)	4.5% (5)	5.9% (2)	6.5% (4)	
Main ICU diagnosis (ICD-10), % (No.)					0.83
I (Cardiovascular disease)	44.2% (50)	36.4% (40)	50.0% (17)	35.5% (22)	
J (Respiratory disease)	14.2% (16)	16.4% (18)	11.8% (4)	9.7% (6)	
U (Other: COVID-19)	6.2% (7)	7.3% (8)	5.9% (2)	9.7% (6)	
C (Neoplasms)	6.2% (7)	6.4% (7)	8.8% (3)	8.1% (5)	
K (Gastrointestinal disease)	4.4% (5)	4.5% (5)	5.9% (2)	11.3% (7)	
Other	24.8% (28)	29.1% (32)	17.6% (6)	25.8% (16)	
Emergency admission, % (No.)	28.3% (32)	31.8% (35)	32.4% (11)	29.0% (18)	0.93
ICU stay (days) (N=312), median (IQR)	6.0 (3.0-15.0)	8.0 (4.0-20.5)	8.5 (5.0-18.0)	9.0 (4.0-19.0)	0.34
SOFA score (N=270), mean (SD)	9.56 (3.93)	9.97 (4.10)	9.39 (3.65)	8.64 (3.40)	0.26
Polypharmacy (≥ 5 medications), % (No.)	56.6% (64)	60.9% (67)	70.6% (24)	53.2% (33)	0.37
PDS-5 score, mean (SD)	20.31 (6.60)	26.65 (7.29)	43.94 (6.73)	49.00 (7.35)	<0.001
PHQ-9 score, mean (SD)	5.48 (2.95)	10.37 (3.32)	11.65 (4.95)	14.40 (3.83)	<0.001
OASIS score, mean (SD)	2.32 (2.14)	8.52 (2.44)	2.68 (2.28)	12.08 (2.35)	<0.001
EQ-5D-5L VAS, mean (SD)	65.59 (18.07)	62.35 (18.86)	53.94 (19.58)	52.74 (19.51)	<0.001
EQ-5D-5L index, mean (SD)	0.81 (0.21)	0.69 (0.28)	0.68 (0.21)	0.60 (0.33)	<0.001
EQ-5D-5L domains, mean (SD)					
Mobility	1.85 (1.24)	2.09 (1.16)	2.06 (1.10)	2.27 (1.23)	0.14
Self-care	1.44 (0.99)	1.52 (0.96)	1.44 (0.79)	1.58 (0.93)	0.80
Usual activities	1.85 (1.22)	2.24 (1.16)	2.18 (1.03)	2.63 (1.03)	<0.001
Pain / Discomfort	2.10 (0.93)	2.40 (1.09)	2.97 (0.87)	2.66 (1.14)	<0.001
Anxiety / Depression	1.65 (0.73)	2.40 (1.03)	2.12 (1.04)	2.98 (1.05)	<0.001

† *Test statistic based on group comparisons appropriate for the variable type and distribution: ANOVA for means, Kruskal-Wallis for medians, and Chi-square for categories. N=319.*

eTable 2. Median Quantile Regression: Determinants of Health-Related Quality of Life

	EQ-5D-5L Index		EQ-5D-5L VAS	
**Variables**	**ß**	**95% CI**	**ß**	**95% CI**
PHQ-9 (z-score)	-0.06***	[-0.09 - -0.03]	-5.73***	[-8.62 - -2.84]
PDS-5 (z-score)	-0.02*	[-0.04 - 0.00]	-3.49**	[-6.26 - -0.72]
OASIS (z-score)	-0.02	[-0.04 - 0.01]	1.08	[-1.60 - 3.76]
Gender (male)	0.05**	[0.00 - 0.09]	-2.99	[-8.04 - 2.05]
Age (years)	-0.00	[-0.00 - 0.00]	-0.13	[-0.32 - 0.06]
Education (CASMIN levels)				
Low	Ref.	-	Ref.	-
Intermediate	0.00	[-0.06 - 0.07]	1.21	[-4.33 - 6.75]
High	0.03	[-0.03 - 0.08]	5.84*	[-0.06 - 11.73]
ICD-10 category, % (No.)				
I (Cardiovascular disease)	Ref.	-	Ref.	-
J (Respiratory disease)	0.02	[-0.05 - 0.09]	0.44	[-5.83 - 6.71]
U (Other: COVID-19)	0.03	[-0.06 - 0.13]	-3.61	[-11.20 - 3.99]
C (Neoplasms)	-0.02	[-0.09 - 0.05]	-2.48	[-11.61 - 6.65]
K (Gastrointestinal disease)	0.03	[-0.06 - 0.11]	-2.58	[-16.77 - 11.61]
Other	-0.04	[-0.10 - 0.01]	-2.62	[-9.35 - 4.10]
Emergency admission	-0.01	[-0.06 - 0.03]	3.15	[-1.92 - 8.23]
SOFA score	-0.01**	[-0.01 - -0.00]	-0.47	[-1.18 - 0.24]
ICU treatment duration (days)	-0.00	[-0.00 - 0.00]	-0.04	[-0.16 - 0.08]
Polypharmacy (>= 5 medications)	-0.05**	[-0.09 - -0.01]	-3.17	[-7.77 - 1.43]

Quantile regression models (τ=0.5) with multiple imputation (m=25) and robust standard errors (N=319).
Coefficients represent differences in the conditional median with 95% confidence intervals in brackets.
**p < 0.10, **p < 0.05, ***p < 0.01.*

eTable 3. Mental Health Impact on Quality of Life – Sensitivity Analysis

	**EQ-5D-5L Index**		**EQ-5D-5L VAS**	
	**Univariate**	**Adjusted†**	**Univariate**	**Adjusted†**
Model 1: Symptom Profiles				
Low symptom burden	Ref.	Ref.	Ref.	Ref.
	-	-	-	-
Anxious-depressive profile	-0.12***	-0.11***	-3.08	-3.16
	[-0.18 - -0.05]	[-0.17 - -0.04]	[-7.97 - 1.81]	[-8.04 - 1.73]
Traumatic-depressive profile	-0.13***	-0.12***	-11.49***	-11.06***
	[-0.21 - -0.05]	[-0.21 - -0.04]	[-18.85 - -4.12]	[-18.51 - -3.61]
High symptom burden	-0.21***	-0.20***	-12.68***	-12.70***
	[-0.30 - -0.12]	[-0.29 - -0.11]	[-18.61 - -6.76]	[-18.64 - -6.76]
Model 2: PHQ-2 Screen (≥ 3 P.)				
PHQ-2 ≥ 3	-0.18***	-0.17***	-11.14***	-10.82***
	[-0.24 - -0.11]	[-0.23 - -0.11]	[-15.32 - -6.97]	[-15.03 - -6.60]

Linear regression models with multiple imputation (m=25) and robust standard errors (N=319); 95% confidence intervals in brackets.
**p < 0.10, **p < 0.05, ***p < 0.01. † Adjusted for sex, age and education.*
